# Operational stability enhancement in organic light-emitting diodes with ultrathin Liq interlayers

**DOI:** 10.1038/srep22463

**Published:** 2016-03-01

**Authors:** Daniel Ping-Kuen Tsang, Chihaya Adachi

**Affiliations:** 1 Center for Organic Photonics and Electronics Research (OPERA), Kyushu University, 744 Motooka, Nishi, Fukuoka 819-0395, Japan; 2 JST, ERATO, Adachi Molecular Exciton Engineering Project, Kyushu University, 744 Motooka, Nishi, Fukuoka 819-0395, Japan

## Abstract

Organic light-emitting diodes (OLEDs) under constant current operation suffer from a decrease of luminance accompanied by an increase of driving voltage. We report a way to greatly improve the stability of OLEDs having a green emitter exhibiting thermally activated delayed fluorescence (TADF), (4s,6s)-2,4,5,6-tetra(9H-carbazol-9-yl) isophthalonitrile (4CzIPN), by introducing ultrathin (1 to 3 nm) interlayers of 8-hydroxyquinolinato lithium (Liq) between hole-blocking layer and its surrounding emissive and electron-transport layers. Under constant current operation starting at a luminescence of 1,000 cd/m^2^, the time to reach 90% of initial luminance (LT_90_) increased eight times, resulting in LT_90_ = 1,380 hours after insertion of the interlayers. Combining this new concept and mixed host system, LT_95_ was further extended to 1315 hours that is 16 times of reference device. This is the best value reported for TADF-based OLEDs and is comparable to the operational lifetimes of well-established phosphorescence-based OLEDs. Thermally stimulated current measurements showed that the number of deep charge traps was reduced with the insertion of the ultrathin Liq interlayer, indicating that reducing the number of deep traps is important for improving the operational lifetime and that exciton-polaron annihilation may be a source of the device degradation.

Organic light-emitting diodes (OLEDs) have been established as a promising technology for highly-efficient solid-state lighting and brilliant displays. For the past 25 years, research groups around the world have been working to enhance the efficiency, turn-on voltage, color purity and stability of OLEDs since the Tang’s seminal report in 1987^1^. In particular, one of the key issues for enhancing OLED performance has been the development of new organic luminescent materials. The performance of OLEDs was significantly enhanced by the jump from first-generation fluorescent emitters to second-generation phosphorescent emitters, realizing nearly 100% internal quantum efficiency (*η*_IQE_) by harvesting triplet excitons for light emission at room temperature^2^. Through the persistent development of organic emitters, third-generation OLED emitters reaching *η*_IQE_ of almost 100% based on thermally activated delayed florescence (TADF) were finally realized in 2012^3^.

Because of their ability to achieve high performance in OLEDs using simple aromatic compounds without a precious metal, TADF emitters have drawn great attention. The key characteristic of efficient TADF emitters is a small energy gap between the lowest singlet-excited and triplet-excited states, allowing efficient up-conversion of triplet excitons into emissive singlet excitons. Through the careful tuning of molecular structures, TADF emitters and OLEDs employing them can obtain nearly 100% upconversion efficiency and top-tier quantum efficiencies^3^. Since the molecular design strategy for TADF emitters provides broad freedom by simply using conventional aromatic moieties, TADF materials can open a wide variety of possibilities in future OLEDs.

Aside from high-performance light-emitting characteristics, long operational lifetime under electrical excitation is necessary for practical applications. In fact, green phosphorescent OLEDs (PHOLEDs) have reached very long operational lifetimes of LT_50_ = 100,000 h, where LT_*x*_ is defined as the time to reach *x*% of the initial luminance *L*_0_ = 1,000 cd/m^2^ under constant current operation^4^. To improve the stability of OLEDs, the development of stable host and emitter materials has been a major focus in the past decade. Another pathway to enhance stability is the development of new OLED device architectures. The insertion of a hole-blocking layer (HBL) or an exciton confinement layer and the utilization of graded doping structures have been shown to effectively improve device lifetime^5^,^6^,^7^,^8^. Among such strategies, the operating lifetime of OLEDs was extended nearly 40% by doping 8-hydroxyquinolatolithium (Liq) into the electron-transporting layers (ETLs)^9^.

Few reports on the enhancement of lifetime in TADF-based OLEDs currently exist. In a previous study, our group studied the relationship between TADF dopant concentration and lifetime and found that LT_50_ = 2,800 h for *L*_0_ = 1,000 cd/m^2^ could be obtained for an emissive layer (EML) of (4s,6s)-2,4,5,6-tetra(9H-carbazol-9-yl)isophthalonitrile (4CzIPN) doped into a 3,3-di(9H-carbazol-9-yl)biphenyl (mCBP) host at a slightly higher doping concentration of 15 wt% compared to the ~5–10 wt% that is commonly used^10^. In this device, an enhancement of electron injection was confirmed when the concentration of dopant was increased, so the longer lifetime was attributed to a shift of the recombination zone toward the center of the EML. However, these lifetimes were still well behind the record values for phosphorescent emitters.

In this work, we found that inserting an ultrathin Liq interlayer between the EML and HBL, as shown in Fig. 1, significantly improves the operational lifetime of 4CzIPN-based OLEDs. By including Liq interlayers with thicknesses of 3 and 2 nm, respectively, at both the EML/HBL and HBL/ETL interfaces, LT_90_ for an initial luminance of 1,000 cd/m^2^ was enhanced eight-fold. The mechanism of the enhancement was investigated by the analysis of the thermally stimulated current (TSC), and the number of deep traps was seen to greatly reduce after the application of the interlayer. This work helps to clarify the device degradation mechanism and dramatically improve OLED lifetime.

The current-density–voltage–luminance (*J*-*V*-*L*) and external quantum efficiency (*η*_EQE_) characteristics of a reference device without Liq and devices A1–A3 with Liq interlayers of 1, 2, and 3 nm, respectively, in position A between the EML and HBL (T2T) are shown in Fig. 2(a,d), and the OLED characteristics are summarized in Table 1. Insertion of the Liq interlayer resulted in an appreciable decrease of luminance and *η*_EQE_, and the *η*_EQE_ values of the reference device and devices A1–A3 were 15.4%, 11.1%, 10.8% and 9.9%, respectively, at 10^−2^ mA/cm^2^. The difference in *η*_EQE_ between the reference and Liq-containing devices is smallest at low current densities (~10^−2^ mA/cm^2^) and grows into a very pronounced gap at higher current densities (>1 mA/cm^2^), suggesting the presence of exciton quenching and/or unbalanced carrier injection and transport in devices A1–A3.

Since excitons primarily form and accumulate near the interface between the EML and HBL, the lower triplet energy (1.96 eV) for Liq (see Experimental) than for mCBP and 4CzIPN could be a possible source of exciton quenching. In fact, the photoluminescence quantum yield (*ϕ*_PL_) of a 15 wt%-4CzIPN: 5 wt%Liq: mCBP film was only 12%, which is much lower than the *ϕ*_PL_ of 90% for a 15 wt%-4CzIPN: mCBP film. In addition, 15 wt% doping of 4CzIPN into an Liq host yielded an even lower *ϕ*_PL_ of 5.5%, indicating that Liq acts as an exciton-quenching agent for 4CzIPN. We also note that increasing the thickness of the Liq interlayer past 3 nm results in an increase in turn-on voltage. While the reference device and device A3 had onset voltages (*V*_on_), defined as the voltage at 1 cd/m^2^, of 3.2 V and 3.8 V, respectively, a 5-nm-thick Liq interlayer at position A resulted in a high *V*_on_ of 5.4 V (Extended Data Fig. 2). Thus, from the aspect of driving voltage, keeping the Liq thickness below 3 nm is preferred. Furthermore, the 5-nm device had a low *η*_EQE_ of ~3%, probably because of unbalanced carrier injection. Although *η*_EQE_ was reduced, the operational lifetimes were greatly improved, as will be discussed later.

For comparison, we fabricated and tested devices with an Liq interlayer of 1, 2, or 3 nm only between the HBL and ETL (position B), which are referred to as devices B1–B3, respectively. As shown in Fig. 2(b,e), the device resistance gradually increased with the thickness of the Liq interlayer. The value of *V*_on_ slightly increased to 3.40 V with the Liq interlayer of 3 nm, while it was 3.20 V in the reference device. Unlike devices A1–A3, there was very little difference in *η*_EQE_ compared with the reference device. Furthermore, an OLED with a 3-nm-thick Liq interlayer inserted between the hole-transporting layer (Tris-PCz) and EML layer was also tested, but resistance dramatically increased, indicating that hole carriers are strongly blocked by the Liq interlayer (Extended Data Fig. 3).

Additionally, an Liq sandwiched structure with Liq interlayers inserted in both positions A and B was examined. Here, the Liq layer at position A was fixed at 3 nm and the thickness of the Liq interlayer at position B was 1, 2, or 3 nm, giving devices C1, C2, and C3, respectively. The OLED properties of devices C1–C3 are shown in Fig. 2(c,f). Like the A and B series of devices, the device resistance increased with the Liq interlayer thickness at position B because of the rather poor electron transporting ability of the Liq layer. Again, we note that *η*_EQE_ in devices C1–C3 showed an appreciable decrease that is similar to devices A1–A3 having a single Liq interlayer in position A.

The emissive spectra were almost identical in all devices as shown in Fig. 3. While a slight redshift in the emission peaks was observed in some devices, the shift was within a few nm, indicating only a very small shift of the emission zones in these devices. There was also a slight increase in the spectra near 450 nm when 3 nm Liq was inserted at position A, which is the emission from Liq.

Figure 4 shows the normalized luminance and change in driving voltage for all of the devices as a function of time operated under a constant current density at room temperature with an initial luminance of 1,000 cd/m^2^. The LT_90_ for the reference device and devices A1–A3 are 175, 485, 630 and 1,115 h, respectively, demonstrating that the lifetime was progressively enhanced with the increase of the Liq interlayer thickness at position A. Device A3, with 3 nm of Liq and the longest LT_90_, exhibited a rather abrupt initial drop in luminance in the first few hours followed by a gradual increase until about 200 h, after which the luminance gradual decreased. On the other hand, in the case of an interlayer inserted at position B, the enhancement of operational stability was only observed when a 1 nm Liq interlayer (device B1) was employed while the other thicknesses resulted in a reduction of device lifetime. The resulting LT_90_ of devices B1–B3 were 290, 140 and 30 hours, respectively.

However, device stability was further enhanced for devices C1–C3. Device C2 with Liq interlayers of 3 nm and 2 nm at positions A and B exhibited nearly an eight-fold increase in LT_90_ over the reference devices, which is the largest enhancement among all of the tested structures, reaching LT_90_ of 1,380 h. Here we again observed the unique luminance drop and increase as seen with device A3, which might be expected since these devices also include 3 nm of Liq at position A. The device luminances rose after their initial decrease during the first ten hours. The peak luminance after the rebound gradually increased when a thicker Liq interlayer was used in position B. The mechanism of the luminance rebound is still unclear, so further investigation is needed. Because Liq could be diffusing into the 10-nm-thick HBL, we also tested the stability of devices with a co-deposited HBL of T2T and Liq. The operational lifetime increased relative to the reference device for Liq concentrations of 25% and 50%, but the enhancement was not as good as the interlayer structures (Extended Data Fig. 5).

Thus, device stability in TADF-based OLEDs could be enhanced almost eight times, with the insertion of an Liq layer between the EML and HBL being the most effective for enhancing device stability. In particular, a 3-nm-thick Liq interlayer in position A resulted in highly stable OLEDs, while some fluctuation in luminance drop and voltage increase were increased from device to device. Since deep traps have been claimed to act as nonradiative recombination centers and play a large role in OLED degradation^11^, we further investigated the degradation mechanism using TSC to probe the traps in the devices.

Typical TSC scans of devices A1–A3 are shown in Fig. 5, with TSC peak temperature reflecting the trap depth (higher temperature corresponds to deeper traps) and peak area reflecting the trap density. From the TSC spectra, shallow (105 K), medium (160 K) and deep (>280 K) traps are evident. The density of shallow traps at around 110 K slightly increased after the insertion of Liq and remained almost constant for the different Liq thicknesses. On the other hand, the deep trap density at >280 K largely decreased as the thickness of Liq increased from 1 nm to 3 nm. Unfortunately, the deep traps cannot be comprehensively measured because the measurement temperature was limited to the glass transition temperature of T2T, *T*_g_ = 55 °C. While the TSC curves in the higher temperature region over 300 K could not be completely characterized, the number of deep traps in device A3 can be presumed to be the lowest among these devices.

Based on these results, we strongly believe that the enhancement of OLED stability can be ascribed to the decrease in initial deep traps. As Giebink *et al.* have demonstrated, deep traps are closely related to PHOLED degradation because they can quench excitons and act as nonradiative charge recombination sites^11^,^12^,^13^. By providing sites for bimolecular exciton-polaron annihilation, deep charge traps can cause degradation when energy transfer from long-lived triplet excitons to the trapped polarons results in the formation of a high-energy polarons that can break the bonds of the molecule on which they reside. From the TSC spectra (Fig. 5), we confirmed that the initial traps inside the devices decreased after the insertion of the Liq interlayer. Thus, the lower deep trap concentration is closely related to the achievement of the longer operational lifetime.

We propose that the formation of deep traps induces exciton-polaron interaction and successive decomposition of organic materials, leading to an increase of driving voltage. In the reference device and devices A1–A3, there is a rather large initial rise in voltage at the beginning of device operation. Within 100 h of operation, the voltages of all of the Liq devices stabilized. After 100 h, the rates of voltage change of devices A1–A3 are much lower values of 0.073, 0.088 and 0.11 mV/h, respectively, compared with the steep increase of 0.51 mV/h in the reference device. Thus, the voltage increase rate was greatly reduced by insertion of an Liq interlayer at position A. On the other hand, the rates of voltage change for devices B1–B3 are 0.37, 0.43, and 0.52 mV/h, respectively, which are close to the value of the reference device. Suppression of the voltage change was again observed in devices C1–C3, indicating that primarily the EML/HBL interface plays an important role for stabilizing the OLEDs.

Possible mechanisms for the lifetime enhancement by the Liq interlayer are as follows. First, we consider triplet exciton quenching by the Liq interlayer. Given the lower triplet energy level and shorter triplet lifetime of an Liq interlayer compared with those of 4CzIPN, the triplet excitons can be quickly quenched through the deactivation channel of Liq. Thus, unwanted triplet exciton-polaron interactions could be suppressed through the internal deactivation channel provided by Liq. To test this idea, we also examined the insertion of ultrathin Alq_3_ and Mg interlayers, which should also act as triplet quenchers. However, both interlayers had no positive effect on the device stability (Extended Data Fig. 6). Thus, other factors should contribute to the enhancement of the device stability.

Secondly, the Liq interlayer may function as an adhesion layer. At the heterointerfaces of organic-organic layers, adjacent hetero-layers may delaminate with the passage of time, which induces local energy barriers for carrier injection, resulting in an increased probability of charge carrier trapping at the interface. Therefore, the ultrathin Liq layer may function as an adhesion layer that helps to maintain good contact for electron injection.

A third possibility is the deactivation of water molecules. It has been well established that a small amount of water molecules are incorporated in organic layers during vacuum deposition of organic layers even under high vacuum conditions^14^. Water molecules would tend to move and localize at the organic heterointerfaces due to hydrophobic effects^15^. Such water might be electrochemically decomposed during OLED operation, resulted in unwanted trap formation. We expect that introduction of an ultrathin Liq layer would deactivate water molecules by coordination with Liq or chemical reactions between water molecules and Liq, leading to the products such as Li(OH).

Last, mCBP host was proven that it is relatively unstable during device operation^16^. Energy barrier of mCBP/T2T and mCBP/Liq are 0.6 eV and 1.1 eV, respectively. Thus, after the insertion of Liq, direct injection of electrons to 4CzIPN guest molecules is more favorable. Device stability enhancement may due to the lack of exciton generation in unstable host, mCBP.

LT_90_ was eight times enhanced after the application of ultrathin Liq sandwich layers, however, the hole leakage into Liq and triplet exciton quenching would inevitably lower the device efficiencies. At 1,000 cd/m^2^, *η*_EQE_ were around 4.4% for C series devices. The lower *η*_EQE_ resulted a relatively high electrical stress towards devices C1–C3. Next, a mixed-host concept was applied in order to control the exciton recombination location, and thus enhanced the *η*_EQE_ of the modified devices. 15% of T2T was doped into EML, which would shift the recombination zone away from the EML/Liq interface due to the enhancement of electron transport ability. A 3 nm thick Liq interlayer was applied at position A only. To further reduce the driving current through the device, Liq was also doped into the electron transporting layer^17^, Bpy-TP2. Devices D1, D2 and D3 corresponded to 0%, 50% and 75% of Liq doped into ETL respectively. Figure 6(a) depicts the *η*_EQE_ characteristics of the devices with various Liq concentrations in the ETL. The *η*_EQE_ values at 1,000 cd/m^2^ of devices D1, D2 and D3 are 6.0%, 6.7% and 6.2%, respectively. All *η*_EQE_ values were increased after the mixed host application, suggesting the shift of the recombination zone towards the center of the EML. As the efficiency enhanced, the driving current was also reduced which leads to a longer device stability. LT_95_ of reference, D1, D2, D3 devices were 80, 610, 935 and 1315 hours respectively as shown in Fig. 6(b). After doping T2T into the EML, the initial drop in luminance as A3 was also disappeared.

We note that an Liq interlayer could also be applied to Ir(ppy)_3_-based PHOLEDs. Extended Data Figure 7 plots the device luminance as a function of operation time for an initial luminance of 1,000 cd/m^2^, showing that the device lifetime was also extended like for 4CzIPN. The same phenomenon observed in PHOLEDs indicates that this technology could be widely applied to a broad variety of OLEDs.

In conclusion, we demonstrated significant enhancement of operational stability in OLEDs by introducing an ultrathin Liq interlayer. The LT_90_ of a 4CzIPN-based OLED with a 3 nm Liq interlayer inserted between the EML and HBL was enhanced by six times, resulting in LT_90_ = 1,115 hours. Further, LT_90_ was enhanced up to 1,380 hours when the HBL was sandwiched on both sides with ultrathin Liq layers. LT_**95**_ was 16 times extended to 1315 hours by the dual application of a mixed host system and an Liq ultra-thin interlayer. This is a very good sign that the TADF molecules, which are composed of electron donating and accepting units, can fundamentally offer stable emission under electrical excitation by designing proper backbone and moieties. While this work helps us to better understand device degradation, the mechanisms need to be further investigated to achieve higher *η*_EQE_ with these long lifetimes. Our next work is to find other suitable materials for the interlayer with the ultimate goal of solving the short lifetime problem of blue TADF-based OLEDs.

## Experimental

### Device fabrication and characterization

Indium tin oxide (ITO) coated glass substrates were used as anodes. Polyimide blank structures were spin coated onto the ITO substrates to make patterns to define active device area of 0.04 cm^2^. TADF-based OLEDs consist of a 10-nm-thick dipyrazino[2,3-f:20,30-h]quinoxaline-2,3,6,7,10,11-hexacarbonitrile (HAT-CN) as a hole injection layer (HIL), a 30-nm-thick 9,99,90-triphenyl-9H,99H,90H-3,39:69,30-tercarbazole (Tris-PCz) as a hole transporting layer (HTL), a 30-nm-thick emissive layer of mCBP doped with 15 wt% of 4CzIPN, a 10-nm-thick 2,4,6-tris(biphenyl-3-yl)-1,3,5-triazine (T2T) as HBL, a 40-nm-thick 2,7-bis(2,20-bipyridine-5-yl)triphenylene (BPy-TP2) as ETL, a 0.8-nm-thick lithium fluoride (LiF) as an electron injection layer (EIL), and a 100-nm-thick aluminum (Al) as metal cathode. HAT-CN, Liq and LiF were used as purchased. Other organic materials were synthesized and purified in our lab. Organic layers were deposited by thermal evaporation under a pressure of 10^–5^ Pa. Various thicknesses of Liq interlayers were deposited and inserted between the EML and HBL and between the HBL and ETL. The deposition rates of the organic and Al layers were 0.1 nm s^–1^, while that of the LiF layer was 0.01 nm s^–1^. Devices were fabricated in different batches, while reference devices were also fabricated in each batch. After fabrication, the devices were immediately encapsulated with glass lids using epoxy glue in nitrogen-filled glove boxes (O_2_, 0.1 ppm, H_2_O, 0.1 ppm). The current density (*J*), voltage (*V*) and luminance (*L*) characteristics of the OLEDs were measured using a Keithley 2400 source meter and an absolute EQE measurement system (C9920-12, Hamamatsu Photonics, Japan). Device operational stability was measured using a luminance meter (CS-2000, Konica Minolta, Japan) at a constant DC current at room temperature.

### Photoluminescence characterization of organic thin films

Organic thin films (100 nm) were firstly deposited on quartz substrates by thermal evaporation under a pressure of 10^–5^ Pa. Photoluminescence spectra were measured by a spectrofluorometer (FluoroMax-4, Horiba Jobin Yvon). Absorption spectra were recorded with an absorption spectrometer (Lamda 950, PerkinElmer). Photoluminescence quantum efficiencies were measured using an absolute PL quantum yield measurement system (C11347-01, Hamamatsu Photonics) under the flow of nitrogen gas with an excitation wavelength of 337 nm.

### TSC measurement

For TSC measurements, the devices were cooled down to liquid-nitrogen temperature and biased at 2 mA/cm^2^ for 2 minutes to charge hole traps with injected holes from the ITO electrode. Then, TSC profiles of the devices were measured during device heating to room temperature with a collecting bias of −0.01 V and a heating rate of 5 °C/min in the TSC measurement chamber (TSC-FETT EL2000, Rigaku). When the devices were not biased at liquid-nitrogen temperature before the heating process, there were no TSC peaks observed from the devices. However, clear TSC peaks were observed after biasing the devices at 2 mA/cm^2^ at liquid-nitrogen temperature, indicating that TSC peaks originated from the de-trapping of holes during the heating process.

### Molecule triplet energy calculation

Quantum chemical calculations were performed using the Gaussian 09 program package^18^. The S0 geometry of Liq were calculated at the B3LYP/6 − 31 + G* level of DFT theory. The electronic energy levels of S1 and T1 were computed at B3LYP/6 − 31 + G* with time-dependent density functional Theory (TD-DFT) using the optimized S0 geometry^19^,^20^.

## Additional Information

**How to cite this article**: Tsang, D. P.-K. and Adachi, C. Operational stability enhancement in organic light-emitting diodes with ultrathin Liq interlayers. *Sci. Rep.* **6**, 22463; doi: 10.1038/srep22463 (2016).

## Supplementary Material

Supplementary Information

## Figures and Tables

**Figure 1:**
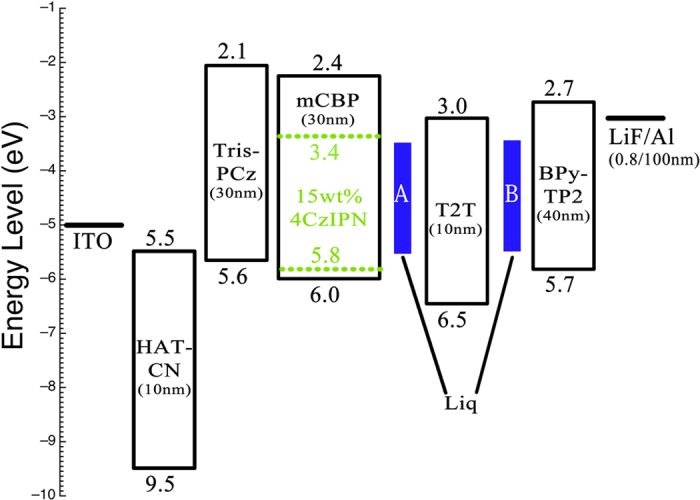
Energy diagram of the fabricated devices. Highest occupied molecular orbital (HOMO) and lowest unoccupied molecular orbital (LUMO) energy levels for the organic compounds and work-functions of the inorganic materials along with the thicknesses of the layers. Energy levels were measured by photoelectron spectroscopy (Riken Keiki, AC-3). E_g_ values were obtained from the absorption edges of films.

**Figure 2:**
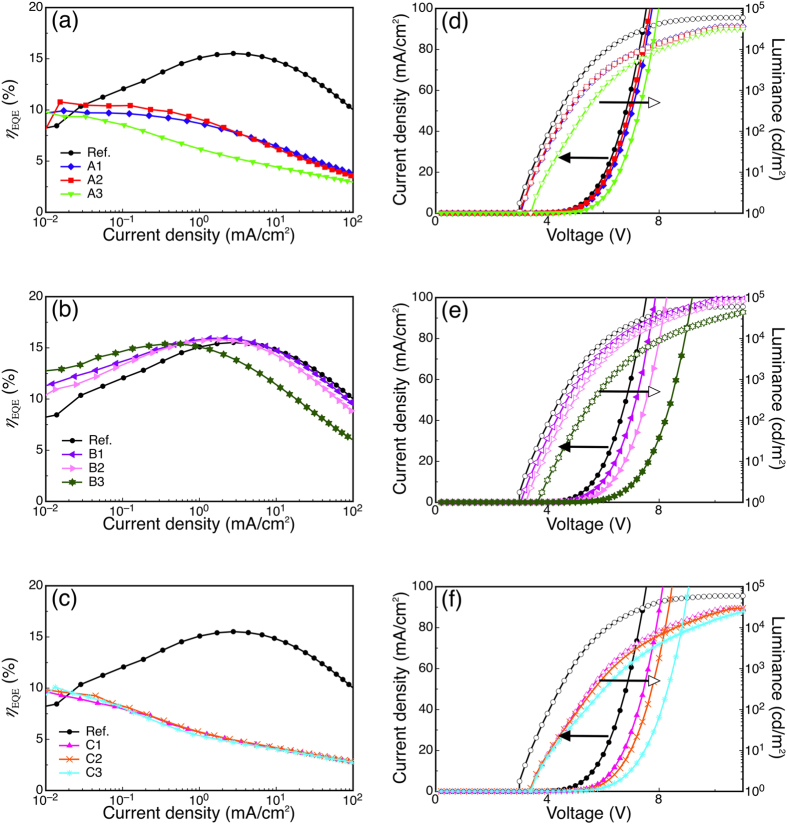
Electrical characteristics of the fabricated devices. (**a–c**) External quantum efficiency vs. current density characteristics for devices with Liq interlayers of 1, 2, or 3 nm between only the EML and HBL (A1–A3), between only the HBL and ETL (B1–B3), or between the HBL and ETL with a 3-nm-thick layer between the EML and HBL (C1–C3). (**d–f**) Luminance (empty symbols) and current-density (filled symbols) as a function of voltage for the devices in (**a–c**).

**Figure 3:**
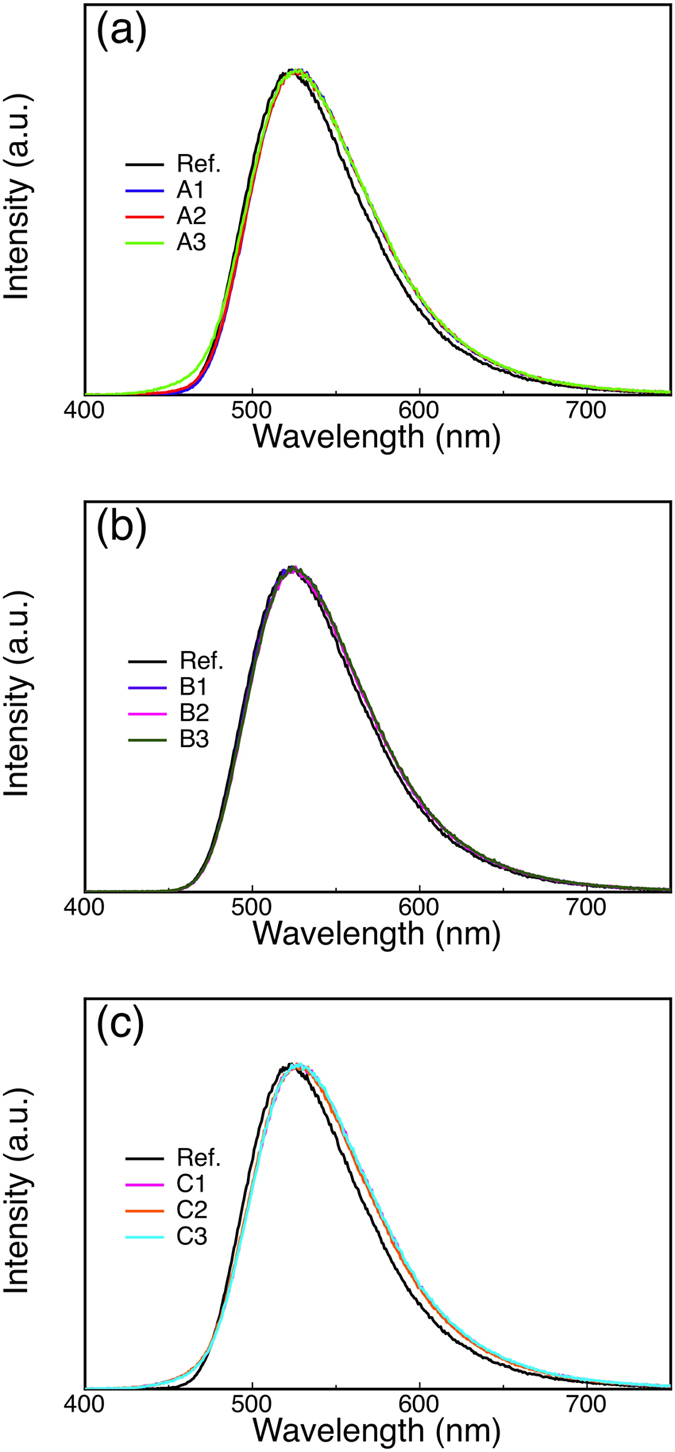
The electroluminescence spectra of the devices at 10 mA/cm^2^. (**a–c**) Normalized electroluminescence spectra of the devices with different Liq configurations and thicknesses as indicated in Table 1.

**Figure 4:**
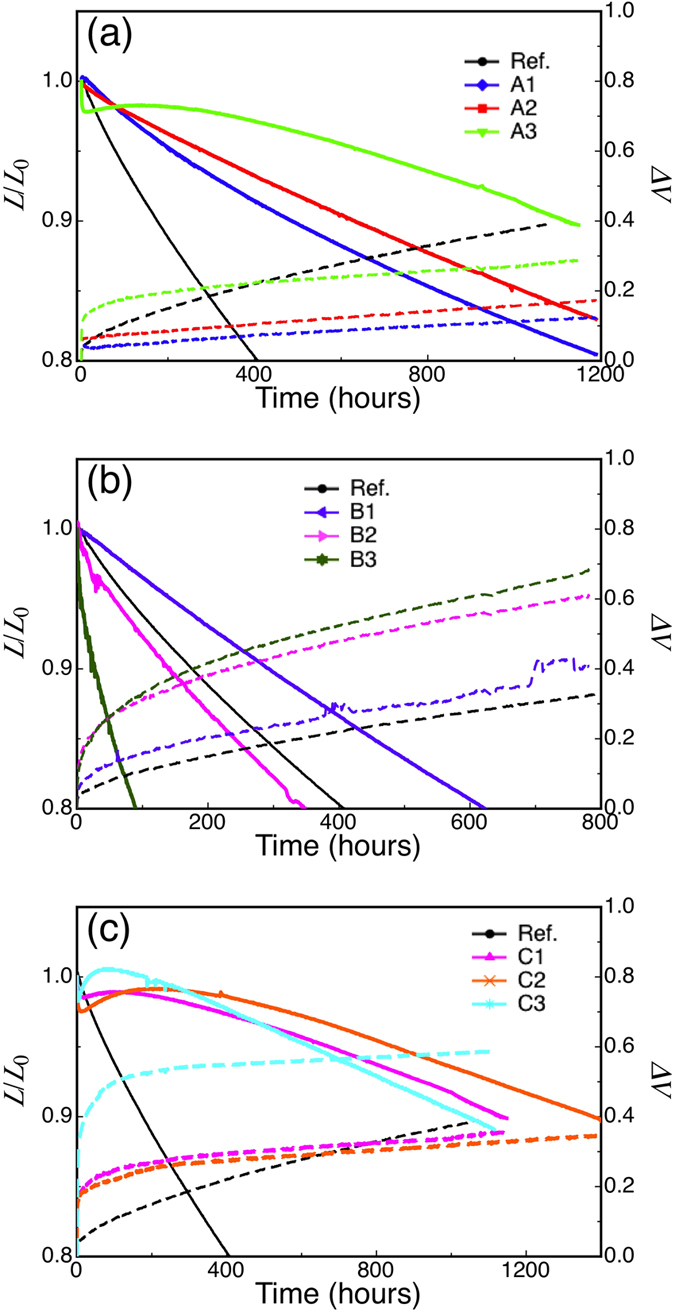
Normalized luminance and change in driving voltage as a function of operating time at a fixed current density with an initial luminance of 1,000 cd/m^2^. (**a–c**) Changes in the luminance (solid lines) and driving voltage (dashed lines) for device series A, B, and C with different Liq interlayer thicknesses (see Table 1).

**Figure 5:**
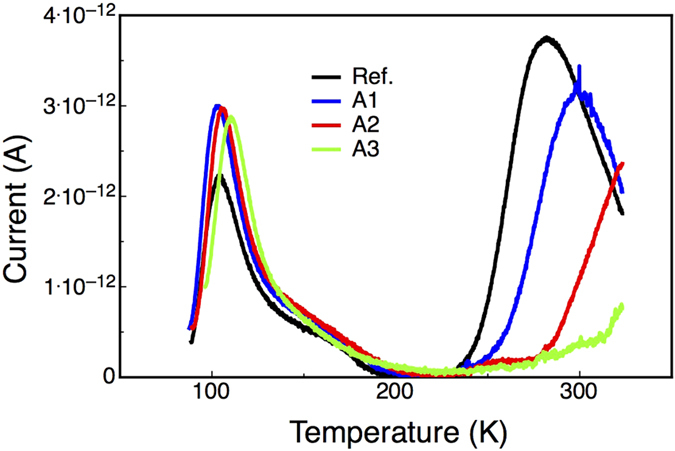
The TSC spectra of 4CzIPN OLED devices with various Liq interlayer thicknesses in position A (reference device and devices A1–A3). No TSC peaks were observed when the devices were not biased at 2 mA/cm^2^ at liquid-nitrogen temperatures before beginning the scan, indicating that the peaks originate from the de-trapping of holes.

**Figure 6:**
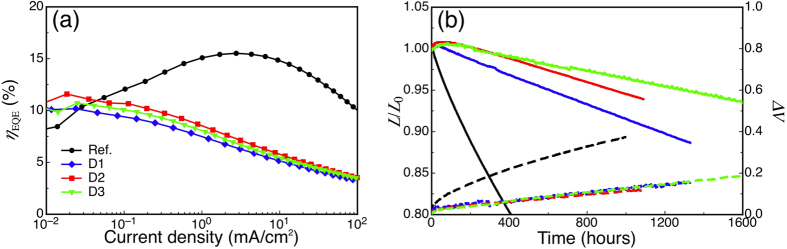
*η*_EQE_ and lifetime characteristics of the mixed-host devices with varies concentration Liq in ETL. (**a**) External quantum efficiency vs. current density characteristics, (**b**) Changes in the luminance (solid lines) and driving voltage (dashed lines) for series D devices with different Liq concentration in ETL (see Table 1).

**Table 1:** Key device characteristics of devices with different Liq interlayer configurations and thicknesses.

Device			Electron transporting layer	Initial characteristics					LT_90_ (h)
	Liq interlayer thickness			Maximum *η*_EQE_(%)	*V*_on_(V)	At 1,000 cd/m^2^			
	Position A (nm)	Position B (nm)				CIE (*x*, *y*)	*η*_EQE_(%)	*J*(mA/cm^2^)	
Ref.	0	0	Bpy-TP2	15.4 ± 0.2	3.2 ± 0.1	0.34, 0.59	15.4 ± 0.2	1.86	175
A1	1	0	Bpy-TP2	11.1 ± 0.1	3.3 ± 0.1	0.34, 0.59	7.5 ± 0.1	4.65	485
A2	2	0	Bpy-TP2	10.8 ± 0.2	3.4 ± 0.1	0.34, 0.58	7.3 ± 0.1	5.57	630
A3	3	0	Bpy-TP2	9.9 ± 0.1	3.8 ± 0.1	0.34, 0.58	4.7 ± 0.1	7.79	1,115
B1	0	1	Bpy-TP2	15.9 ± 0.2	3.0 ± 0.1	0.34, 0.59	15.9 ± 0.1	1.98	290
B2	0	2	Bpy-TP2	15.8 ± 0.1	3.2 ± 0.1	0.34, 0.59	15.8 ± 0.1	2.00	140
B3	0	3	Bpy-TP2	15.4 ± 0.2	3.4 ± 0.1	0.34, 0.59	14.0 ± 0.2	2.10	30
C1	3	1	Bpy-TP2	9.8 ± 0.1	3.8 ± 0.1	0.34, 0.58	4.4 ± 0.1	7.04	1,130
C2	3	2	Bpy-TP2	9.9 ± 0.1	3.8 ± 0.1	0.34, 0.58	4.4 ± 0.1	6.81	1,380
C3	3	3	Bpy-TP2	10.0 ± 0.1	3.8 ± 0.1	0.34, 0.58	4.2 ± 0.1	7.11	1,045
D1	3	0	Bpy-TP2	11.2 ± 0.1	3.4 ± 0.1	0.34, 0.58	6.0 ± 0.1	4.75	1200
D2	3	0	50%Liq : Bpy-TP2	11.6 ± 0.2	3.2 ± 0.1	0.34, 0.58	6.7 ± 0.1	4.33	935 (LT_95_)
D3	3	0	75% Liq : Bpy-TP2	11.3 ± 0.1	3.2 ± 0.1	0.34, 0.58	6.2 ± 0.1	3.83	1315 (LT_95_)

*Maximum *η*_EQE_ and *V*_on_ denote the maximum external quantum efficiency and the voltage at 1 cd/m^2^. Values were averaged and standard deviations calculated from at least four devices. Here, CIE refers to Commission Internationale de l’Eclairage coordinates. Operational lifetimes were measured with *L*_0_ = 1,000 cd/m^2^.
