# Corrigendum: Operational stability enhancement in organic light-emitting diodes with ultrathin Liq interlayers

**DOI:** 10.1038/srep26921

**Published:** 2016-05-31

**Authors:** Daniel Ping-Kuen Tsang, Toshinori Matsushima, Chihaya Adachi

Scientific Reports 6: Article number: 2246310.1038/srep22463; published online: 03 01 2016; updated: 05 31 2016

Toshinori Matsushima was omitted from the author list in the original version of this Article. This has been corrected in the PDF and HTML versions of the Article.

The Acknowledgements section now reads:

This work was supported by the Exploratory Research for Advanced Technology (ERATO) and the Kumamoto Collaborations on Organic Electronics under the Regional Innovation Strategy Support Program sponsored by the Ministry of Education, Culture, Sports, Science and Technology (MEXT) of Japan. We would like to acknowledge Mr. Ko Inada for his assistance with calculation of molecular energy levels. We also thank Dr. William J. Potscavage Jr. for his assistance with the preparation of this manuscript.

The Author Contributions section now reads:

D.T. performed the OLED and lifetime experiments. T.M. performed the TSC measurements and analysis. D.T. and C.A. wrote the paper with input from T.M. C.A. supervised the project.

