# Supplementary material for: Operational stability enhancement in organic light-emitting diodes with ultrathin Liq interlayers
Source: Sci Rep. 2016 Mar 1;6:22463. doi: 10.1038/srep22463 (PMC4772539; doi:10.1038/srep22463)
Supplement: Supplementary Information [file srep22463-s1.pdf]

Extended Data

Operational stability enhancement in organic light emitting diodes with ultrathin Liq interlayers

[1]Daniel Ping-Kuen Tsang and [1,2]Chihaya Adachi

[1]Center for Organic Photonics and Electronics Research (OPERA), Kyushu University, 744 Motooka, Nishi, Fukuoka 819-0395, Japan

[2]JST, ERATO, Adachi Molecular Exciton Engineering Project, Kyushu University, 744 Motooka, Nishi, Fukuoka 819-0395, Japan

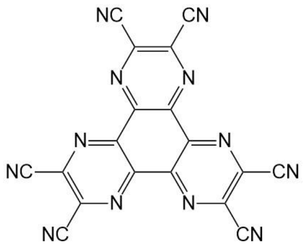

HAT-CN

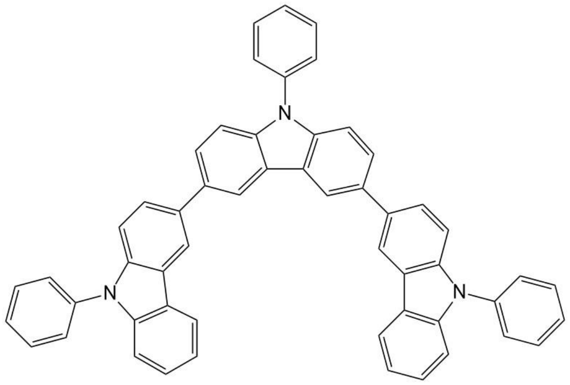

Tris-PCz

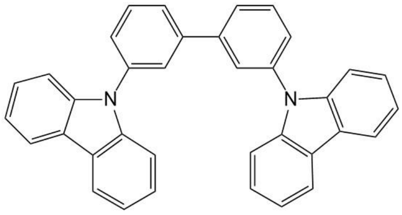

mCBP

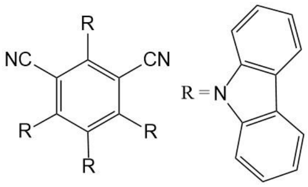

4CzIPN

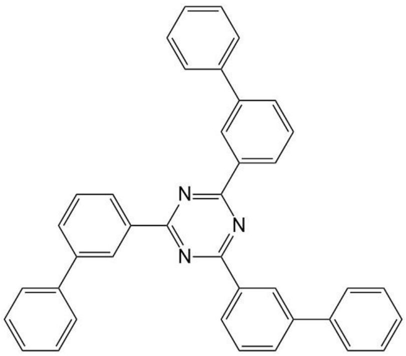

T2T

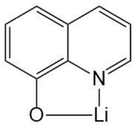

Liq

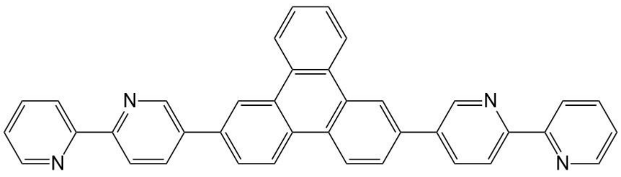

BPy-TP2

Extended Data Figure 1 | Chemical structures of materials in the tested devices.

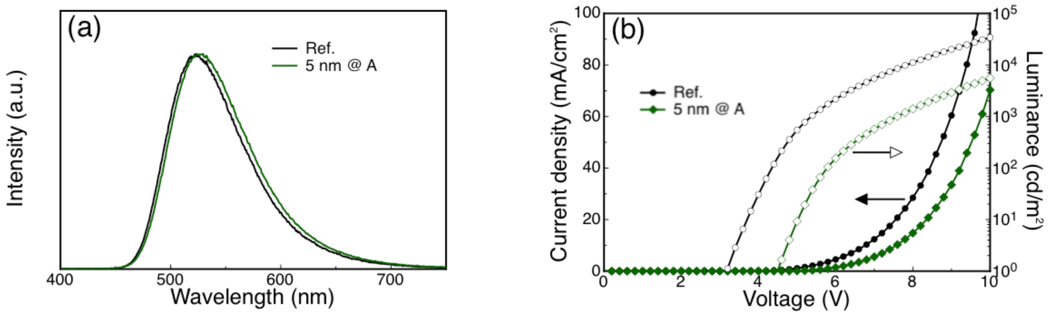

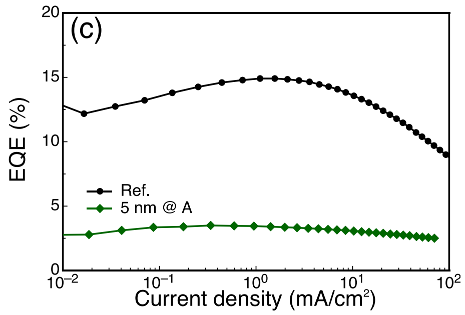

Extended Data Figure 2 | The electroluminescence characteristics of the reference device (Ref.) and a device with a 5-nm-thick Liq interlayer at position A. a, Electroluminescence spectra at 10 mA/cm^2. **b,** Luminance (empty symbols) and current density (filled symbols) as a function of voltage. **c,** External quantum efficiency vs. current density characteristics.

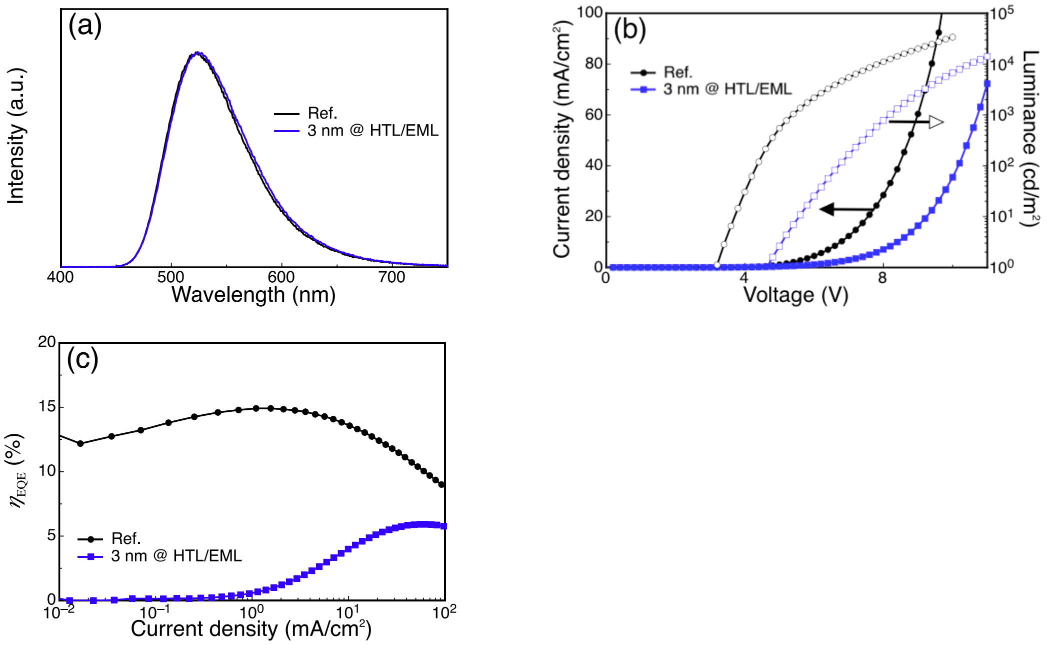

Extended Data Figure 3 | The electroluminescence characteristics of the reference device (Ref.) and a device with a 3-nm-thick Liq interlayer between the HTL and EML. a, Electroluminescence spectra at 10 mA/cm^2. **b,** Luminance (empty symbols) and current density (filled symbols) as a function of voltage. **c,** External quantum efficiency vs. current density characteristics.

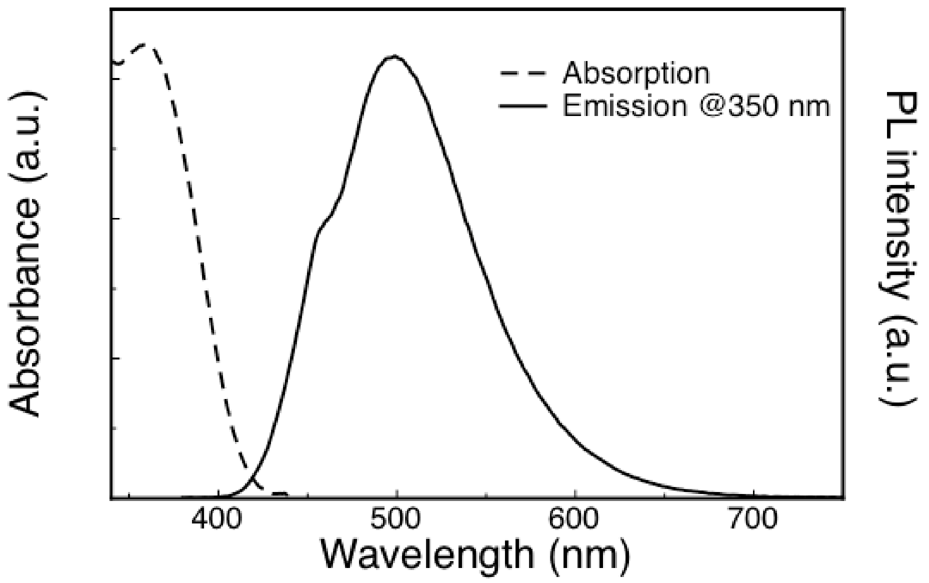

Extended Data Figure 4 | The absorption and photoluminescence spectra of Liq film excited by a 350 nm light source.

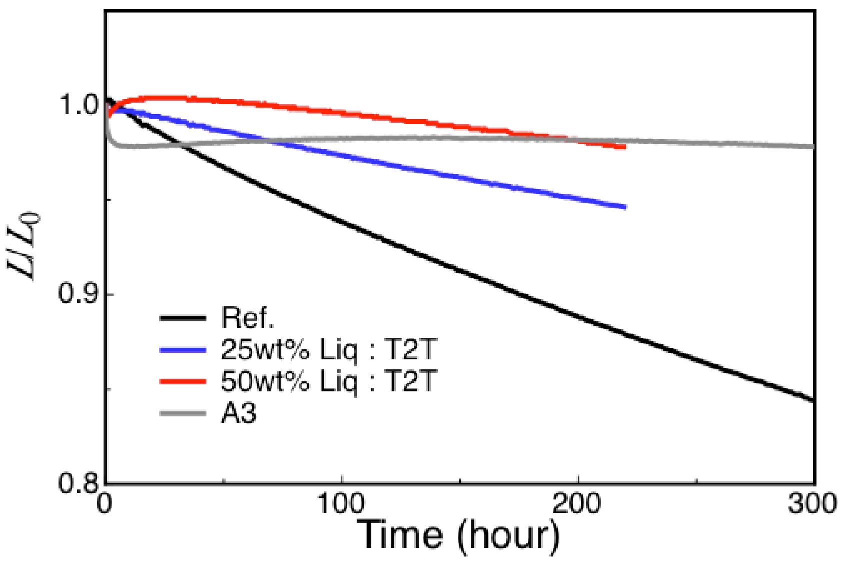

Extended Data Figure 5 | Luminance vs. operating time at a fixed current density with an initial luminescence of 1,000 cd/m^2 for the reference device (Ref.) and devices with an HBL doped with Liq. The structure of the devices with the doped HBML was ITO / HAT-CN (10 nm) / Tris-PCz (30 nm) / 15 wt% 4CzIPN:mCBP (30 nm) / 25wt% Liq:T2T (blue) or 50wt% Liq:HBL (red) / Bpy-TP2 (40 nm) / LiF (0.8 nm) / Al (100 nm).

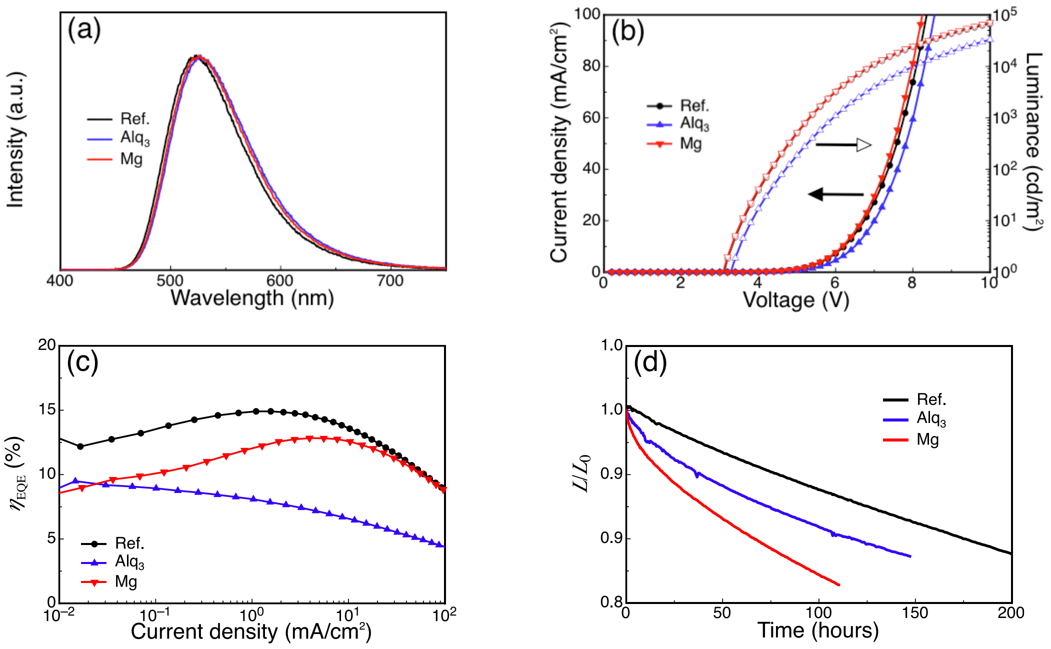

Extended Data Figure 6 | The electroluminescence characteristics of a reference device (Ref.) and devices with a 3-nm-thick interlayer of either 3 nm Alq_3 or Mg at position A. a, Electroluminescence spectra at 10 mA/cm^2. **b,** Luminance (empty symbols) and current density (filled symbols) as a function of voltage. **c,** External quantum efficiency vs. current density characteristics. **d,** Luminance vs. operating time at a fixed current density with an initial luminescence of 1,000 cd/m^2.

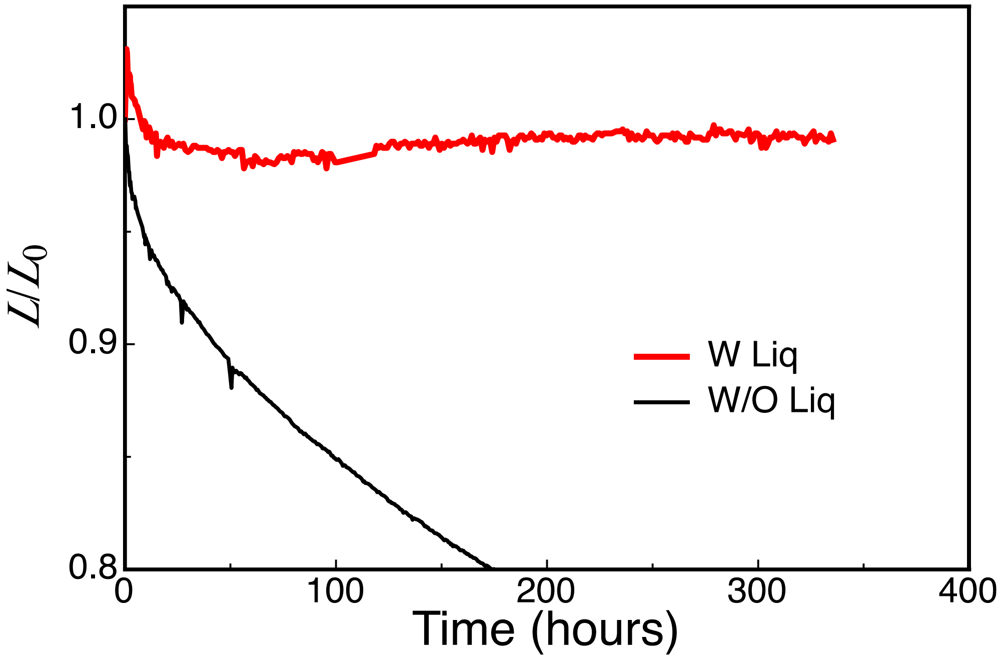

Extended Data Figure 7 | Luminance vs. operating time at a fixed current density with an initial luminescence of 1,000 cd/m^2 for phosphorescent OLEDs with and without Liq. The device structure is ITO / HAT-CN (10 nm) / Tris-PCz (30 nm) / 8 wt% Ir(ppy)$_3$:mCBP (30 nm) / (red curve only) Liq (3 nm) / T2T (10 nm) / Bpy-TP2 (40 nm) / LiF (0.8 nm) / Al (100 nm).
